# Neurologic Safety of Etomidate-Based Sedation during Upper Endoscopy in Patients with Liver Cirrhosis Compared with Propofol: A Double-Blind, Randomized Controlled Trial

**DOI:** 10.3390/jcm9082424

**Published:** 2020-07-29

**Authors:** Jang Han Jung, Bomi Hyun, Jin Lee, Dong Hee Koh, Jung Hee Kim, Se Woo Park

**Affiliations:** Division of Gastroenterology, Department of Internal Medicine, Hallym University Dongtan Sacred Heart Hospital, Hallym University College of Medicine, Hwaseong-si 18450, Gyeonggi-do, Korea; con2000@hallym.or.kr (J.H.J.); hyunbom@hallym.or.kr (B.H.); jinlee@hallym.or.kr (J.L.); dhkoh@hallym.or.kr (D.H.K.); jungheekim@hallym.or.kr (J.H.K.)

**Keywords:** safety, etomidate, propofol, liver cirrhosis, encephalopathy

## Abstract

(1) Background: Although etomidate-based sedation is an effective and safe protocol in endoscopic procedures, there is a lack of evidence regarding the safety of etomidate in patients with liver cirrhosis (LC). This study aimed to compare the neurologic safety and efficacy of etomidate and propofol for endoscopic sedation in patients with LC. (2) Methods: From December 2017 to December 2019, consecutive cirrhotic patients who underwent sedative endoscopy using either etomidate or propofol were randomly recruited. The primary endpoint was the number connection test (NCT), and the secondary endpoints included factors for the safety of sedatives during endoscopy. (3) Results: 63 patients were enrolled in each of the etomidate and propofol groups. The NCT times were significantly lower in the etomidate group than in the propofol group. Furthermore, severe or very severe degree of encephalopathy was higher in the propofol group but was not significantly different. Pharmacological properties and the overall incidence of respiratory and cardiovascular events did not differ significantly between the groups. (4) Conclusion: Etomidate-based sedation exacerbates neither subclinical nor overt hepatic encephalopathy. It guarantees efficacies similar to those of propofol regarding rapid sedation, fast recovery, and early discharge, with no increased risk of adverse respiratory or cardiovascular events in patients with LC.

## 1. Introduction

Patients with compensated or decompensated liver cirrhosis (LC) are usually referred to diagnostic or therapeutic endoscopy to be screened for varices and portal hypertensive gastropathy, or endoscopic bleeding control [1,2]. To enhance patient tolerance and satisfaction, diagnostic endoscopy is frequently conducted under sedation [3], even in cirrhotic patients. In addition, sedation is usually administered in interventional endoscopy. However, patients with hepatic dysfunction who undergo endoscopic sedation could be at increased risk of adverse events related to sedation. Among these adverse events is the exacerbation of minimal hepatic encephalopathy (MHE), which is a major concern in these patients. Furthermore, because many drugs are metabolized in the liver, sedative drugs can lead to a risk of excessive and prolonged drug effect due to higher plasma concentration and delayed clearance [4]. Several recent studies [5,6,7] have demonstrated that sedation with propofol led to a shorter recovery time and earlier discharge than midazolam and did not exacerbate MHE in patients with LC. Although propofol also is metabolized in the liver, no adjustment or reduction of drug dose is necessary in patients with LC [8] and its use may be reasonable in these patients [9]. However, potential risk of adverse events, including neurologic, respiratory, and cardiovascular could occur during sedation with propofol, especially in those patients with LC who have decreased effective arterial blood volume in spite of increased plasma and blood volume [10]. Most studies [11,12,13] reported that the use of propofol during endoscopy for patients with LC is safe, since it does not lead to acute deterioration in MHE. However, the mean number connection test (NCT) score for propofol-based sedation was 87.5 (±62) s, with the median NCT score indicating a moderate degree of encephalopathy. Therefore, propofol-based sedation in patients with LC may have limited utility in clinical practice, despite its previously reported advantages.

Our two recent randomized trials [14,15] demonstrated that etomidate can be a reasonable sedative option in countries where an anesthesiologist is not available during endoscopic interventions. Moreover, etomidate had a lower risk of respiratory and cardiovascular adverse events while inducing deep sedation. Thus, etomidate showed a superior safety profile even in patients at risk of acute cardiovascular instability with a guarantee of at least the same drug profile as in other alternative drugs such as propofol.

Considering these previous reports on the safety of etomidate, it might have the potential to replace propofol as an endoscopic sedative in patients with LC. However, whether etomidate results in superior outcomes compared to these of propofol for sedative endoscopy in patients with LC, especially with regard to MHE, remains unclear. Thus, we designed a prospective randomized controlled study to compare the effects of etomidate-based versus propofol-based endoscopic sedation on neurologic and cardiovascular adverse events in patients with LC. 

## 2. Methods

### 2.1. Study Design and Population 

This study was a single-center, double-blind, randomized controlled trial conducted between December 2017 and December 2019. This study included all patients aged 18–80 years with chronic liver disease who had evidence of LC by clinical, laboratory, and/or pathologic criteria (Child–Pugh class A, B, or C) and were undergoing diagnostic or therapeutic upper gastrointestinal (GI) endoscopy (UGIE) [16] at our medical center. All patients were evaluated regarding the current MHE based on clinical history and neurologic examination. Patients were excluded for any of the following criteria: (a) clinically detectable hepatic encephalopathy, psychiatric illness, mental impairment, or active neurological impairment; (b) recent administration of neuro-active drugs that might interfere with etomidate or propofol metabolism; (c) a history of prior adverse events with sedative agents; (d) known allergy to egg products, tofu, soy beans, propofol, or etomidate; (e) known adrenocortical insufficiency, long-term steroid therapy, or porphyria; (f) renal impairment with serum creatinine >2 mg/dL; (g) breast-feeding or pregnant; (h) willingness to have endoscopy without sedation; and (i) refusal to participate in the study or provide informed consent. Before procedure, the patients dropped out of the trial if their mean blood pressure (MBP) was <60 mmHg (or systolic blood pressure (SBP) <90 mmHg and/or diastolic blood pressure <50 mmHg) or oxygen saturation (SpO2) was <90% breathing room air or <95% with 2 L/min oxygen.

### 2.2. Randomization

All patients who met the inclusion criteria underwent diagnostic or therapeutic UGIE in random sequence assigned to either the etomidate or propofol group in a 1:1 ratio using a table of computer-generated random numbers created by one of the authors (J.H.K.). The allocation sequence, according to the random table, was concealed using opaque sealed envelopes, and neither the authors nor patients were aware of treatment allocation. In addition, since both etomidate and propofol are milk-like whitish solutions, they could not be distinguished visually.

### 2.3. Sedation Protocols 

All physicians and nurses administering sedatives followed the recommendations for clinical practice guidelines [17,18]. Furthermore, they were trained to deal with advanced cardiac life support, including the monitoring of vital signs and essential pharmacological theory. One experienced endoscopist, one trainee endoscopist, and two nurses were involved in the procedure. A committed investigator has kept monitoring patient’s vital signs independently from any endoscopic procedure or other situations. One other nurse and a trainee endoscopist assisted in the procedure and a float nurse also was on standby for emergency situations. All procedures were conducted in an endoscopy room with a full set of equipment; oropharyngeal airways, face mask, laryngoscope, endotracheal tube, stylet, ambu-bag, defibrillator, and drugs for immediate resuscitation. Patients were monitored with continuous pulse oxymeter oxygen saturation (SpO_2_) monitor, electrocardiography, visual inspection of respiration via thoracic cage, and intermittent blood pressure measurements every 5 min. During the procedure, nasal supplemental oxygen (2 L/min) was given to all patients. The target level of sedation was deep level, meaning that the patient had a response to only painful stimulation while keeping stable vital signs, as defined by a score of 0–2 on the Modified Observer’s Assessment of Alertness/Sedation (MOAA/S) scale [17].

In both groups, before the study drug (either etomidate or propofol) was administered, one 100 μg fentanyl buccal tablet was given for the prevention of myoclonus as an adverse event of study drug administration. In the etomidate group, an initial bolus of 0.05 mg/kg (0.025 mL/kg) etomidate (Etomidate Lipuro, 20 mg/10 mL/Ampoule, B.Braun Korea, Seoul, Korea) was administered. In the propofol group, an initial bolus of 0.25 mg/kg (0.025 mL/kg) propofol (Fresofol MCT, 150 mg/15 mL/Ampoule, Fresenius-Kabi Korea, Seoul, Korea) was administered [14]. Undersedated patients, with an MOAA/S score of 3–6, received repeated doses of either etomidate (0.05 mg/kg; 0.025 mL/kg; etomidate group) or propofol (0.25 mg/kg; 0.025 mL/kg; propofol group) to maintain the target level of sedation. An additional bolus of sedatives was administered, at the same volume in both groups, after at least 1 min of observation to assess the complete effect of the drug. Administering the same volume of sedative ensured investigators remained blinded to group allocation [14].

### 2.4. Survey of Patients’ Satisfaction with Sedation

After full recovery of sedation, patients gave their satisfaction scores for sedation on a verbally categorized scale of 1 to 4; 1 as excellent, 2 as good, 3 as fair, and 4 as poor. The point of memory during the procedure of patients and intention to repeat endoscopic procedure under the same conditions were also assessed using questionnaires. 

### 2.5. Endoscopic Procedure and Assessment of Hepatic Encephalopathy

Following sedation, all endoscopic procedures were performed according to the standard techniques. After procedure, patients were closely monitored in the recovery room by another independent nurse who made a judgment of recovery assessment. The nurse documented the time of leaving the endoscopy room, and the time at which patients were judged to have fully recovered [19]. The NCT (Trail Making Test A), which is defined as the time required to connect consecutive number placed randomly from 1 to 25, was also conducted [5]. The degree of encephalopathy, defined according to protocols outlined previously [20], was scored as 0 (none) if patients had a completion of test within 15–30 s, 1+ (mild) within 31–50 s, 2+ (moderate) within 51–80 s, 3+ (severe) within 81–120 s, and 4+ (very severe) if unable to complete the test within 120 s [5]. 

### 2.6. Study Endpoint

A psychometric test was the primary endpoint (the NCT). Furthermore, the following secondary endpoints were assessed: (a) incidence of each adverse event, including hypoxemia, respiratory depression, apnea, hypotension, and arrhythmia, (b) incidence of myoclonus or a paradoxical reaction, (c) the administered dose of sedatives, and (d) other changes in vital signs (heart rate (HR), SBP, MBP, and SpO_2_) during sedation.

### 2.7. Definitions

A major adverse event was defined as endotracheal intubation, permanent neurological impairment, or death. Adverse cardiovascular events included bradycardia, tachycardia, arrhythmia, and hypotension. Bradycardia and tachycardia were defined as a HR <60 beats/min or >100 beats/min, respectively. Arrhythmia was defined as any irregular rhythm deviating from a normal sinus rhythm. Furthermore, hypotension was defined as SBP <90 mmHg or decline >20 mmHg from the baseline [8]. A respiratory event was defined as desaturation with SpO_2_ <90% or when airway intervention including jaw-thrust/chin-lift maneuver, an increase in O_2_ flow, and assisted ventilation was required.

Induction time was defined as the time interval between initial administration of the sedative and insertion of the endoscope. Total procedure time was defined as the time interval between the insertion and withdrawal of the endoscope. Awake time was defined as the time interval between the withdrawal of the endoscope and the full recovery of the patient (Aldrete score of 10) [21]. Finally, recovery time was defined as the time interval between the withdrawal of the endoscope and patient discharge from the endoscopy unit. 

### 2.8. Statistical Analysis

To calculate the sample size, we referred to a recent study [5] using the same sedation protocol with propofol (intermittent manually administration) for endoscopy in cirrhotic patients, in which the mean time for the completion of the NCT in the propofol group was 74.2 (± 58.0) s in patients with LC undergoing endoscopy. To achieve a power of at least 80% and detect 30 s of absolute difference between the two groups with an alpha level of 0.05, the number of samples required was 59 patients per group. Assuming a 5% dropout rate, the final sample size was therefore set at 63 patients per group. 

Descriptive statistics are provided for categorical and continuous variables, with number/proportion or mean (standard deviation, SD), respectively. Chi-square or Fisher’s exact tests were used to compare categorical variables, and a two-sample t-test was used to compare continuous variables. A *p*-value of <0.05 was considered statistically significant. All statistical analyses were conducted using the statistical software R (version 3.2.3; R Foundation for Statistical Computing, Vienna, Austria). 

### 2.9. Ethics Statement

The study protocol was approved by the institutional review board of the Hallym University College of Medicine Dongtan Sacred Heart Hospital, Korea on 13 November 2017 (HDT 2017-09-006-003) before the initiation of the study. The study was conducted in accordance with good clinical practice guidelines, the Declaration of Helsinki, and the Health Insurance Portability and Accountability Act. All patients provided written informed consent before enrollment. This trial is registered with the International Clinical Trials Registry Platform, no. (KCT0004904).

## 3. Results

**Figure 1:**
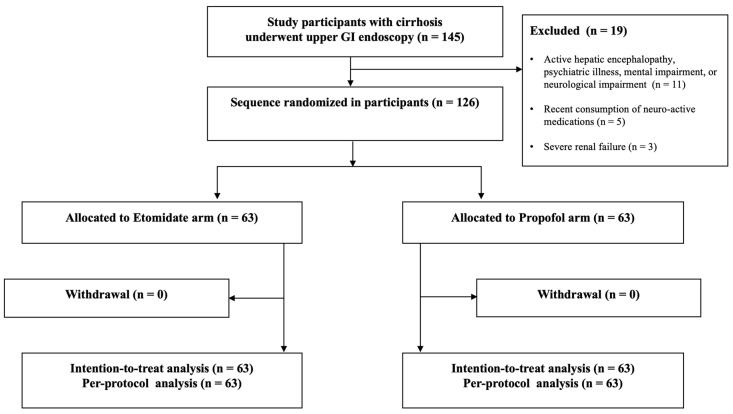
Flow chart showing patient allocation and progression throughout the randomized trial. GI, gastrointestinal; SaO_2_, arterial oxygen saturation.

A total of 145 cirrhotic patients who underwent UGIE were initially assessed for eligibility (Figure 1). Out of these, patients were excluded because of a clinically detectable hepatic encephalopathy, psychiatric illness, mental impairment, or active neurological impairment (*n* = 11), recent consumption of neuro-active drugs (*n* = 5), or renal impairment with serum creatinine >2 mg/dL (*n* = 3). As a result, the remaining 126 patients were randomly assigned to either the etomidate (*n* = 63) or propofol (*n* = 63) group. After randomization, all 126 patients received their allocated intervention, and none were lost during follow-up or excluded from analysis.

### 3.1. Baseline Patient Characteristics 

The mean patient age was 54.2 and 55.6 years in the etomidate and propofol groups, respectively (*p* = 0.492; Table 1). The proportion of male patients was higher in the etomidate group, although not significantly so (etomidate vs. propofol, 73.0% vs. 68.3%; *p* = 0.696). In addition, there were no between-group differences in body mass index, smoking history, alcohol intake, or alcohol intake amounts. In both groups, alcohol was the most common etiology for LC (etomidate vs. propofol, 84.1% vs. 84.1%; *p* > 0.999). In addition, baseline liver function, as measured by Child–Turcotte–Pugh (CTP) and Model for End-stage Liver Disease (MELD) scores, did not differ between the etomidate and propofol groups (CTP, 7.3 (1.9) vs. 7.1 (1.9); *p* = 0.602; MELD, 13.7 (9.4) vs. 14.6 (9.5); *p* = 0.594).

### 3.2. Procedures and Sedation-Related Outcomes 

In both groups, screening purpose was the most common indication for endoscopy (etomidate vs. propofol, 77.8% vs. 82.5%; Table 2). In addition, interventional endoscopy procedures such as endoscopic variceal ligation (EVL) or endoscopic injection sclerotherapy (EIS) were performed in six cases in each group, and the most common source of bleeding in both groups was an esophageal varix. Furthermore, the grade of varices did not differ between the groups. 

The procedure time and induction time were shorter in the etomidate group than these in the propofol group, although not significantly so (procedure time, 7.1 [3.6] vs. 8.6 [10.0] min, *p* = 0.242; induction time, 110.2 [94.0] vs. 122.5 [65.0] s, *p* = 0.392). In contrast, the awake time and recovery time were longer in the etomidate group than these in the propofol group (awake time, 12.4 [7.8] vs. 10.7 [6.4] min, *p* = 0.181; recovery time, 22.9 [8.9] vs. 20.4 [8.2], *p* = 0.114), but not significantly different. The administered doses were 0.043 (0.039) mg∙min^−1^kg^−1^ of etomidate and 0.256 (0.174) mg∙min^−1^ kg^−1^ of propofol. 

### 3.3. Sedation-Related Adverse Events and Satisfaction 

No major adverse events occurred in either group. Overall respiratory adverse events were identified in three patients (4.8%) in the etomidate group and four patients (6.3%) in the propofol group (*p* > 0.999; Table 3). In particular, desaturation defined as SpO_2_ < 90% did not differ between the groups (etomidate vs. propofol, 4.8% vs. 6.3%; *p* > 0.999), and only one patient with apnea required airway intervention in the etomidate group. There was no between-group difference in the incidence of cardiovascular adverse events (etomidate vs. propofol, 41.3% vs. 34.9%, *p* = 0.435). Specifically, there was no significant between-group difference in the incidence of tachycardia or bradycardia (etomidate vs. propofol: tachycardia, 27.0% vs. 22.2%, *p* = 0.679; bradycardia, 6.3% vs. 11.1%, *p* = 0.528). Furthermore, the incidence of transient hypotension with an SBP below 90 mmHg did not differ between groups (etomidate vs. propofol, 12.7% vs. 7.9%, *p* = 0.558). Among the adverse events that interfered with the procedure, paradoxical reactions requiring physical restraint did not differ between the groups (etomidate vs. propofol, 4.8% vs. 6.3%; *p* > 0.999), although the incidence of myoclonus tended to be higher in the etomidate group than in the propofol group (19.0% vs. 6.3%, *p* = 0.061). There was a significant between-group difference in NCT results (etomidate vs. propofol, 54.1 [43.8] s vs. 85.1 [111.9] s; *p* = 0.044). Furthermore, a severe or very severe degree of encephalopathy was more frequent in the propofol group, but not significantly different. In both groups, the majority of patients were satisfied with the sedation during the procedure (etomidate vs. propofol: satisfaction score, 9.1 [1.5] vs. 9.0 [1.4], *p* = 0.854; Excellent, 54.0% vs. 49.2%; Good, 41.3% vs. 47.6%; Fair, 3.2% vs. 1.6%; Poor, 1.6% vs. 1.6%, *p* = 0.860). Overall, all satisfaction-related outcomes were similar between the groups. 

### 3.4. Changes in Vital Signs over Elapsed Sedation Time 

**Figure 2:**
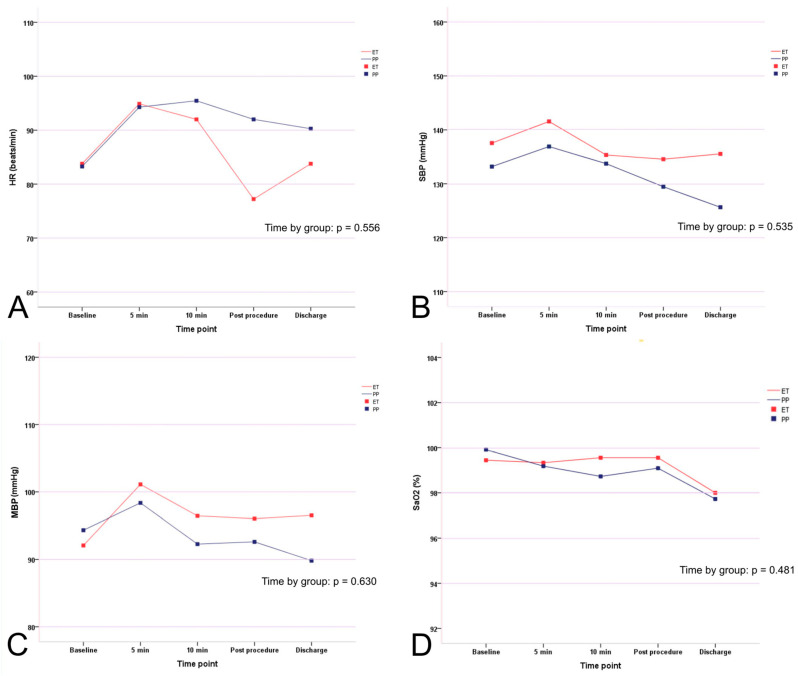
Time course showing changes in heart rate, blood pressure, and oxygen saturation. Mean values of (**A**) heart rate (beats/min), (**B**) systolic blood pressure (mmHg), (**C**) mean blood pressure (mmHg), and (**D**) arterial oxygen saturation (%), at baseline (at least 15 min before the initial drug administration), 5 and 10 min after initiating sedation during endoscopy, awake time, and at discharge from the endoscopy unit.

HR, SBP, and MBP peaked at 5 min after starting sedation (except for HR in the propofol group, which peaked after 10 min) and decreased thereafter (Figure 2). However, the group-by-time interaction was not significant for HR (*p* = 0.556), SBP (*p* = 0.535), MBP (*p* = 0.630), or SaO_2_ (*p* = 0.481), indicating that the sedative agents did not affect HR, blood pressure, or arterial oxygen saturation patterns during the study period (Figure 2).

## 4. Discussion

This study aimed to evaluate neurologic outcomes in patients with LC undergoing etomidate-based and propofol-based sedation during endoscopy. Our results indicate that the sedative effects of etomidate with regard to pharmacological properties were similar to those of propofol. Although induction time, recovery time, and patient satisfaction did not differ between the groups, etomidate proved to be a favorable agent to achieve safe and effective sedation in patients with LC with regard to cognitive function. Conversely, it took longer for patients in the propofol group to complete the NCT after an endoscopic procedure than those in the etomidate group. This suggests the possibility of MHE, i.e., patients who apparently show relatively normal mental status on clinical test but exhibit neurological impairment when undergoing neuropsychological tests [22,23]. Our results are clinically relevant because they showed that etomidate-based sedation results in faster and safer procedures and less co-morbidity.

Many studies [11,12,13] reported the neurologic safety of propofol during UGIE in patients with LC, and that does not lead to acute deterioration in MHE. In addition, propofol induces and maintains sedation and amnesia by interacting with the GABA receptors, which leads to immediate onsets and offsets, even after prolonged infusion [9]. Thus, propofol is associated with shorter recovery times even in patients with LC than midazolam does [24,25]. Furthermore, a recent meta-analysis demonstrated that propofol-based sedation during endoscopy causes no impairment in psychometric scores, such as the NCT A and B; the digit symbol test; line tracing test; and serial dotting tests [8]. In another study, the mean NCT time for propofol was 87.5 (± 62) s with a median NCT score indicating a moderate encephalopathy grade, which accords with our results [5]. Although there was no data with regard to the pharmacological properties of etomidate in patients with LC, or comparative results with propofol, subjects receiving etomidate took significantly less time to complete NCTs, with similar induction, awake, and recovery times. A unique study reporting pharmacokinetic data of etomidate in patients with LC demonstrated that clearance values of etomidate in LC were no different from those in healthy individuals, although wide volume distribution resulting in the prolongation of terminal half-life was observed in decompensated LC patients [26]. Moreover, the pharmacological properties of etomidate that we assessed, including induction, awake, and recovery times, were comparable with our recent studies for non-cirrhotic patients [14,15].

One of our significant findings is the relatively low incidence of myoclonus in etomidate-based sedation (around 19%) compared to 20% to 45% reported previously [27]. This discrepancy may relate to the fact that we premedicated patients with fentanyl buccal tablets before the administration of etomidate. Regarding other respiratory and cardiovascular adverse events, including desaturation, shock, and arrhythmia, our data shows that their incidence was not significantly different between the etomidate and propofol groups. This might explain that etomidate-based sedation did not increase the risk of respiratory and cardiovascular adverse events in patients with LC.

Although our present study is the first to evaluate the neurologic safety of etomidate-based sedation compared to propofol in patients with LC, it has several limitations. Firstly, we did not measure the baseline NCT before the administration of sedative agents for endoscopy. However, previous studies [6,28] have shown a learning effect associated with repetitive psychometry tests and some studies [29] have consequently indicated that any impairment in performing these psychometry tests would be obscured by their repetition. Additionally, there was insufficient time to measure NCT before emergency hemostatic interventions, especially in patients with acute variceal bleeding. Secondly, we measured neurologic outcomes based on a single abnormal psychometry test (NCT-A), whilst Ferenci et al. [22] have recommended that two or more tests are necessary to diagnose MHE. Among these, Critical Flicker Frequency as a language-independent tool has an excellent specificity and sensitivity for diagnosing MHE and could be a reasonable choice of psychometric testing [30]. Nevertheless, among the many psychometric tests available, the NCT is considered the standard in this area because of its sensitivity, ease of application, and quantitative aspect [31,32]. Thirdly, the patients’ serum cortisol levels were not assessed in the groups, either before or after procedures. Although, even a single and low dose of etomidate can result in adrenal impairment, it appears to be transient and has not been associated with prolonged adrenal insufficiency or even increased mortality [14,15]. Furthermore, we have already excluded the patients with a known adrenocortical insufficiency or porphyria, as well as those who underwent long-term steroid therapy before study enrollment. Although etomidate-based sedation is shown to be safe in the field of endoscopy, the use of etomidate under a non-anesthesiologist is strictly prohibited in some countries, including the United States and Europe. It can, however, be an effective and safe sedative option in other countries [33], especially South Korea [34], where propofol-based sedation by non-anesthesiologists is restricted. Finally, the NCT measurement was not uniformly determined at the same time point across the patients in both groups. It could lead to subjective bias, although the awake time varies from person to person according to total dosage of sedative agents and body surface area.

Recently, a study has been published to measure spleen stiffness to reduce sedative endoscopy for screening purposes rather than sedative endoscopy for bleeding and other therapeutic purposes [35]. Developing a predictive model that can identify patients requiring sedative endoscopy may be clinically helpful in patients with high hepatic encephalopathy risks, as well as areas with high barriers to access to endoscopic procedures for reasons such as medical costs or medical facilities.

Despite these limitations, our study revealed the efficacy and neurologic safety of etomidate-based sedation in patients with LC. Etomidate-based sedation does not exacerbate MHE and guarantees a similar efficacy to propofol, with regard to rapid sedation, fast recovery, and early discharge, and with no increased risk of respiratory or cardiovascular adverse events in patients with LC.

## Figures and Tables

**Table 1:** Baseline patient characteristics.

Variable	Etomidate (*n* = 63)	Propofol (*n* = 63)	*p*-Value
Age, year, mean ± SD	54.2 ± 10.8	55.6 ± 11.7	0.492
Sex (Male), *n* (%)	46 (73.0%)	43 (68.3%)	0.696
Body mass index, kg·m^2^, mean ± SD	23.5 ± 4.0	23.1 ± 3.3	0.504
Smoking cigarette, *n* (%)	34 (54.0%)	33 (52.4%)	>0.999
Alcohol intake, *n* (%)	49 (77.8%)	51 (81.0%)	0.826
Amounts of alcohol, g/week, mean ± SD	360 ± 285	360 ± 345	0.992
Etiology of liver disease, *n* (%)			
HBV	9 (14.3%)	10 (15.9%)	>0.999
HCV	2 (3.2%)	2 (3.2%)	>0.999
Alcohol	53 (84.1%)	53 (84.1%)	>0.999
Others	2 (3.2%)	2 (3.2%)	>0.999
History of hepatic encephalopathy, *n* (%)	9 (14.3%)	9 (14.3%)	>0.999
Child Turcotte Pugh, *n* (%)	7.3 ± 1.9	7.1 ± 1.9	0.602
A	24 (38.1%)	27 (42.9%)	0.773
B	30 (47.6%)	26 (41.3%)	
C	9 (14.3%)	10 (15.9%)	
MELD, *n* (%)	13.7 ± 9.4	14.6 ± 9.5	0.594

Values are expressed as mean ± SD or number of the patients (%).

**Table 2:** Procedure and sedation-related outcomes.

Variable	Etomidate (*n* = 63)	Propofol (*n* = 63)	*p*-Value
Indication of endoscopy, *n* (%)			0.677
Emergency EVL or EIS or other bleeding control	6 (9.5%)	6 (9.5%)	
Scheduled EVL or EIS	8 (12.7%)	5 (7.9%)	
Screening	49 (77.8%)	52 (82.5%)	
Bleeding source, *n* (%)			>0.999
Esophageal varix	5 (7.9%)	5 (7.9%)	
Gastric varix	1 (1.6%)	1 (1.6%)	
Endoscopic classification			0.214
Active variceal bleeding at endoscopy	2 (3.2%)	5 (7.9%)	
High risk stigmata	4 (6.3%)	1 (1.6%)	
Grade of varices			0.947
F0	18 (28.6%)	21 (33.3%)	
F1	13 (20.6%)	12 (19.0%)	
F2	22 (34.9%)	20 (31.7%)	
F3	10 (15.9%)	10 (15.9%)	
Induction time, sec, mean ± SD	110.2 ± 94.0	122.5 ± 65.0	0.392
Procedure time, min, mean ± SD	7.1 ± 3.6	8.6 ± 10.0	0.242
Awake time, min, mean ± SD	12.4 ± 7.8	10.7 ± 6.4	0.181
Recovery time, min, mean ± SD	22.9 ± 8.9	20.4 ± 8.2	0.114
Administered dose of sedatives			
Fentanyl, ug	100	100	>0.999
Etomidate, ml, mean ± SD	7.9 ± 3.9		
Etomidate, mg, mean ± SD	15.8 ± 7.7		
Etomidate, mg/min/kg, mean ± SD	0.043 ± 0.039		
Propofol, ml, mean ± SD		10.1 ± 5.1	
Propofol, mg, mean ± SD		101.0 ± 50.7	
Propofol, mg/min/kg, mean ± SD		0.256 ± 0.174	

Values are expressed as mean ± SD or number of the patients (%).

**Table 3:** Adverse event and satisfaction for sedation.

Variable	Etomidate (*n* = 63)	Propofol (*n* = 63)	*p*-Value
Adverse event, *n* (%)			
Overall respiratory event	3 (4.8%)	4 (6.3%)	>0.999
Desaturation ^1^	3 (4.8%)	4 (6.3%)	>0.999
Apnea required airway intervention ^2^	1 (1.6%)	0 (0.0%)	>0.999
Overall cardiovascular event	26 (41.3%)	22 (34.9%)	0.435
Tachycardia ^3^	17 (27.0%)	14 (22.2%)	0.679
Bradycardia ^4^	2 (3.2%)	7 (11.1%)	0.166
Shock ^5^	8 (12.7%)	5 (7.9%)	0.558
Overall event that interfered with procedure	11 (17.2)	14 (22.2)	0.476
Myoclonus	12 (19.0%)	4 (6.3%)	0.061
Paradoxical reaction required physical restraint ^6^	3 (4.8%)	4 (6.3%)	>0.999
Psychometric test (Number connection test), s	54.1 ± 43.8	85.1 ± 111.9	0.044
None	24 (38.1%)	15 (23.8%)	0.247
Mild	10 (15.9%)	14 (22.2%)	
Moderate	18 (28.6%)	15 (23.8%)	
Severe	8 (12.7%)	12 (19.0%)	
Very severe	3 (4.8%)	7 (11.1%)	
Satisfaction for sedation of patient	9.1 ± 1.5	9.0 ± 1.4	0.854
Satisfaction for sedation, *n* (%)			
Patient			0.860
Excellent	34 (54.0%)	31 (49.2%)	
Good	26 (41.3%)	30 (47.6%)	
Fair	2 (3.2%)	1 (1.6%)	
Poor	1 (1.6%)	1 (1.6%)	
Recall, *n* (%)			
At endoscope insertion	13 (20.6%)	8 (12.7%)	0.339
During the procedure	1 (1.6%)	2 (3.2%)	>0.999
At endoscope withdrawal	4 (6.3%)	5 (7.9%)	>0.999
At leaving the operating room	11 (17.5%)	11 (17.5%)	>0.999
Willingness to repeat endoscopic procedure with the same conditions, *n* (%)	52 (82.5%)	55 (87.3%)	0.619

Values are expressed as mean ± SD or number of the patients (%). ^1^ Oxygen saturation [SpO_2_] to less than 90%. ^2^ The cessation of respiratory airflow or need for bag mask or mechanical ventilation. ^3^ Heart rate more than 100 beats per minute. ^4^ Heart rate less than 50 beats per minute. ^5^ Systolic blood pressure < 90 mmHg. ^6^ Restraint of the patient beyond holding of the hands longer than 3 s during the procedure.
